# 26Postoperative diagnosis and outcome in patients with revision arthroplasty for aseptic loosening

**DOI:** 10.1186/s12879-015-0976-y

**Published:** 2015-06-18

**Authors:** Marta Fernandez-Sampedro, Carlos Salas-Venero, Concepción Fariñas-Álvarez, Manuel Sumillera, Luis Pérez-Carro, Michel Fakkas-Fernandez, Javier Gómez-Román, Luis Martínez-Martínez, María Carmen Fariñas

**Affiliations:** Infectious Diseases Unit, Hospital Universitario Marqués de Valdecilla, University of Cantabria, Avenida de Valdecilla s/n, Santander, 39008 Spain; Department of Microbiology, Hospital Universitario Marqués de Valdecilla, University of Cantabria, Santander, Spain; Health Care Quality Unit, Hospital Universitario Marqués de Valdecilla, University of Cantabria, Santander, Spain; Department of Traumatology and Orthopaedic Surgery, Hospital Universitario Marqués de Valdecilla, University of Cantabria, Santander, Spain; Department of Pathology, Hospital Universitario Marqués de Valdecilla, University of Cantabria, Santander, Spain

**Keywords:** Prosthetic joint infection, Aseptic loosening, Implant failure

## Abstract

**Background:**

The most common cause of implant failure is aseptic loosening (AL), followed by prosthetic joint infection (PJI). This study evaluates the incidence of PJI among patients operated with suspected AL and whether the diagnosis of PJI was predictive of subsequent implant failure including re-infection, at 2 years of follow up.

**Methods:**

Patients undergoing revision hip or knee arthroplasty due to presumed AL from February 2009 to September 2011 were prospectively evaluated. A sonication fluid of prosthesis and tissue samples for microbiology and histopathology at the time of the surgery were collected. Implant failure include recurrent or persistent infection, reoperation for any reason or need for chronic antibiotic suppression.

**Results:**

Of 198 patients with pre-and intraoperative diagnosis of AL, 24 (12.1 %) had postoperative diagnosis of PJI. After a follow up of 31 months (IQR: 21 to 38 months), 9 (37.5 %) of 24 patients in the PJI group had implant failure compared to only 1 (1.1 %) in the 198 of AL group (*p* < 0.0001). Sensitivity of sonicate fluid culture (>20 CFU) and peri-prosthetic tissue culture were 87.5 % *vs* 66.7 %, respectively. Specificities were 100 % for both techniques (95 % CI, 97.9–100 %). A greater number of patients with PJI (79.1 %) had previous partial arthroplasty revisions than those patients in the AL group (56.9 %) (*p* = 0.04). In addition, 5 (55.5 %) patients with PJI and implant failure had more revision arthroplasties during the first year after the last implant placement than those patients with PJI without implant failure (1 patient; 6.7 %) (RR 3.8; 95 % CI 1.4-10.1; *p* = 0.015). On the other hand, 6 (25 %) patients finally diagnosed of PJI were initially diagnosed of AL in the first year after primary arthroplasty, whereas it was only 16 (9.2 %) patients in the group of true AL (RR 2.7; 95 % CI 1.2–6.1; *p* = 0.03).

**Conclusions:**

More than one tenth of patients with suspected AL are misdiagnosed PJI. Positive histology and positive peri-implant tissue and sonicate fluid cultures are highly predictive of implant failure in patients with PJI. Patients with greater number of partial hip revisions for a presumed AL had more risk of PJI. Early loosening is more often caused by hidden PJI than late loosening.

## Background

Infection is a challenging problem associated with orthopedic implants. It is projected in the United States that the percentages of deep implant infections for hip and knee arthroplasties will increase from 8.4 to 47.5 % and from 16.8 to 65.5 %, respectively, through 2030 [1, 2]. Currently, prosthetic joint infection (PJI) is the second most common cause of implant failure [3]. However, infection rates are probably underestimated, since many cases of presumed aseptic failure may be due to unrecognized infection.

The pathogenesis of aseptic loosening (AL) in prosthetic joints is not well understood, and several lines of evidence suggest that occult bacteria may cause some AL cases. First using a sonication method to remove biofilm bacteria from AL of orthopedic implants, 3 studies have reported positive sonicate-fluid cultures in 9.5, 11.2 and 57.7 % of the patients, respectively [4–6]; secondly an association has been found between the degree of osteolysis and sonication cultures in patients with AL of hip and knee implants [6]; and third, the ability of bacterial subpopulations to switch to strains resulting in an unusual morphological appearance called small-colony variants (SCVs), which often remain undetected [7].

The aim of this study was to evaluate the incidence of PJI among patients with suspected AL, assessing whether postoperative diagnosis of PJI in the revision arthroplasty by histological and microbiological analysis of the periprosthetic tissues and sonicate fluid, was predictive of implant failure including re-infection, at 2-year follow-up.

## Methods

The study was conducted in the Division of Orthopaedics of the Hospital Universitario Marques of Valdecilla (a 900-bed tertiary health care hospital), which performs approximately 500 and 300 hip knee arthroplasties per year. A prospective cohort study from February 2009 to September 2011, of all consecutive patients who underwent one-stage partial or total hip or knee revision arthroplasty because of presumed AL, was performed. Patients >18 years old, without prior documented history of infection in the index prosthesis since the primary surgery of knee or hip arthroplasty were included. A diagnostic preoperative algorithm was applied to all patients to accurately determine the cause of the prosthesis failure and rule out infection. The algorithm included a history and physical examination, determination of erythrocyte sedimentation rate (ESR) and C-reactive protein (CRP) within 2 weeks before surgery, a plain radiograph and a technetium-99-labeled leukocyte scintigraphy [8]. Pre-operative synovial fluid culture was obtained at the surgeon’s discretion. Patients were excluded if there had fewer than 2 peri-implant tissue samples collected, the implant was not obtained or obvious contamination of the implant was identified in the operating room o histology examination was not performed.

Data (demographics, comorbid conditions, type of implant, surgical procedure, antimicrobial treatment and outcome), were prospectively collected by the clinical research associates from the institution using a standardized data collection form. Patients received standard peri-operative prophylaxis with cefazolin or vancomycin in cases of penicillin allergy; which was continued for 24 hours after surgery. All surgeries were performed in a standard, non-laminar airflow operating room. The choice of a surgical strategy for revision arthroplasty was at the surgeon’s discretion and type and duration of antibiotic therapy was at the discretion of the patient’s physician.

Patients were following up after the inclusion in the study for at least 24 months. Clinical outcomes include recurrent or persistent infection, reoperation for any reason, need for chronic antibiotic suppression. The functional status includes the assessment of painful prosthesis and describes symptomatic (pain) or asymptomatic (pain-free) patients.

### Definitions

- **Pre-operative diagnosis of AL** was established when the patient had local pain, radiological signs of loosening without clinical symptoms (fever) or signs of infection (hyperthermia, swelling, hydrops, rubor, sinus tract), the CRP or ESR levels were normal and technetium-99-labeled leukocyte scintigraphy and pre-operative synovial fluid culture (when performed) were negative.
- **Intraoperative diagnosis of AL** was established in the absence of visible purulence in the synovial fluid or surrounding the prosthesis or sinus tract communicating with the prosthesis.
- **Postoperative diagnosis of AL** was established as implant failure that did not meet criteria for diagnosis of PJI.
- **Diagnosis of PJI** was defined as previously described by Del Pozo et al. [9]: 1) acute inflammation was detected on histopathological examination of periprosthetic tissue or 2) isolation of the same organism from at least 2 periprosthetic tissue cultures, isolation of the organism ≥ 20 colony-forming units (CFU) on either plate from the sonicate fluid, or both. When *S. aureus* or *S. lugdunensis* were the microorganisms isolated, only a single positive tissue specimen or ≥1 CFU on sonicate fluid culture plate was required.
- **Implant failure** includes recurrent or persistent infection, re-operation for any reason or need for chronic antibiotic suppression.

#### Specimen collection, cultures and sonication

Intraoperatively, peri-implant tissue samples with the most obvious inflammatory changes were collected for histopathology and conventional microbiologic culture. Two to 6 peri-implant tissue specimens per patient were collected. Removed orthopedic implants, cultures and prosthesis sonication were performed as previously described [10, 11].

#### Statistical analysis

The baseline characteristics of patients classified within AL and PJI groups were compared using the ANOVA test for continuous variables or the chisquared test or Fisher’s exact test for categorical variables. The sensitivity and specificity of the different culture methods were compared using McNemar’s test of paired proportions. Ninety-five percent confidence intervals (95 % CI) were calculated as exact binomial confidence intervals. The diagnostic accuracy of sonicate fluid cultures were evaluated by constructing a receiver-operating-characteristic (ROC) curve. Relative risks (RR) and their confidence intervals (CI) to 95 % were calculated. A p-value of less than 0.05 (two-sided) was considered statistically significant. Calculations were performed with the SPSS package v19.0 (SPSS Inc., Chicago, Illinois), and the Stata statistical software (Release 10.0, Stata Corporation, College Station, TX).

### Ethics

All patients sign the inform consent to participate in the study. The study protocol was approved by the Ethics Committee of the Autonomous Community of Cantabria (IFIMAV, Spain) and the Fondo de Investigaciones Sanitarias [Registered number: FIS PI080609].

## Results

### Study population

**Fig. 1:**
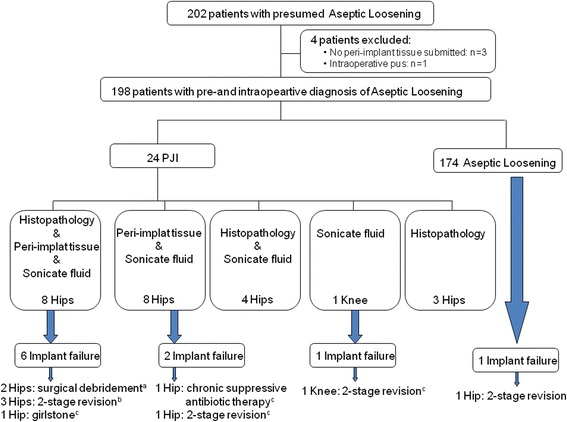
Distribution of study patients. **a** One out of 2 cases received antibiotic therapy post-revision arthroplasty. **b** One out of 3 cases received antibiotic therapy post-revision arthroplasty. **c** Received antibiotic therapy post-revision arthroplasty

A total of 202 patients with presumed AL were included in the study. Four patients (1.5 %) were excluded: 3 because no peri-implant tissue was submitted to microbiology laboratory and 1 because PJI diagnose was made intraoperatively. Of the remaining 198 patients with pre-and intraoperative diagnosis of AL (141 hips and 57 knees), 174 had AL and 24 had a diagnosis of PJI. Three out of the 24 PJI cases were diagnosed by histopathological findings; 8 had histopathology and positive periprosthetic tissue and sonicate fluid cultures; 8 had positive periprosthetic tissue and sonicate fluid cultures; 4 had histopathology and positive sonicate fluid cultures; and 1 had positive sonicate fluid culture (Fig. 1). All study patients were followed-up for a median time of 31 months (interquartile range [IQR]: 21 to 38 months) after revision arthroplasty.

The demographic and clinical characteristics of patients with AL and PJI are shown in Table 1. Both groups (patients with AL and PJI) had similar age, gender, comorbidities (mostly Diabetes), underlying joint disorders (mainly Ostheoarthrosis and Osteoporosis), prior revision arthroplasty, time between last implantation and removal of implant and length of surgery. There was higher percentage of hip prosthesis in the group with PJI than in the AL: 95.8 % *vs* 67.8 %; *p* = 0.03. Patients with PJI had a greater number of previous partial revisions than the AL group: 79.1 % *vs* 56.9 %; *p* = 0.04.

**Table 1:** Demographic and clinical characteristics of study patients

Characteristic	Aseptic loosening (*n* = 174)	PJI (*n* = 24)	*P* ^a^
Age (year)-mean (SD)	69.5 (12.0)	68.2 (14.1)	0.61
Gender- no. (%)			
Male	72 (41.4)	14 (58.3)	0.13
Body mass index-mean (SD)	30.8 (4.8)	30.6 (6.1)	0.84
Location			
Hip	118(67.8)	23(95.8)	0.003
Knee	56 (32.2)	1(4.2)	
Surgical procedure-no. (%)^a^			
Partial Revision	99 (56.9)	19 (79.1)	0.045
Total Revision (One-stage exchange)	73 (42.0)	4 (16.7)	
Two-stage revision	1 (0.6)	0	
Girdlestone	1 (0.6)	1(4.2)	
Underlying joint disorder-no (%)			
Osteoarthritis	6 (3.4)	0	1.00
Osteoarthrosis	14 (8.0)	4 (16.7)	0.24
Osteoporosis	19 (10.9)	4 (16.7)	0.49
Rheumatoid arthritis	7 (4.0)	0	1.00
Acute fracture/trauma	9 (5.2)	1(4.2)	1.00
Malignancy	3 (1.7)	0	1.00
Associated conditions- no. (%)			
Diabetes mellitus	25 (14.4)	5 (20.8)	0.38
Immunosuppressive therapy	6 (3.4)	0	1.00
Systemic steroid therapy	6 (3.4)	0	1.00
Prior revision arthroplasty	34 (19.5)	8 (33.3)	0.18
Time between last implantation and removal of implant-no. (%)			
≤30 days	1 (0.6)	1 (4.2)	0.12
31– 365 days	15 (8.6)	5 (20.8)	
366 days-5 years	51 (29.3)	6 (25.0)	
6-10 years	32 (18.4)	5 (20.8)	
11-15 years	31 (17.8)	5 (20.8)	
>15 years	44 (25.3)	2 (8.3)	
Blood units after surgery-no. (%)	32 (18.4)	8 (33.3)	0.11
Length of surgery (minutes)-	152.6 (70.7)	173.8 (93.9)	0.20392
Mean (SD)			393

*PJI* Prosthetic joint infection; *SD* Standard Deviation

^a^Two-tailed Chi-squared or Fisher exact test for categorical variables or t-test for continuous variables

### Microbiology cultures

Two to 6 peri-implant tissue samples were collected per patient in the 198 patients of the study (mean 3.27samples per patient). The distribution of the tissue samples was as follow: in 4 patients 6 samples (2 %) were collected; in 12 patients 5 samples (6 %), in 34 patients 4 samples (17.3 %); in 138 patients 3 samples (69.7 %) and in 10 patients 2 samples (5 %). There were 11 AL cases in which only a single peri-implant was positive culture and, no one of these patients meet any other criteria for PJI. Futhermore, all of them had 3 or more peri-implant tissue samples collected (in 2 cases 5 samples; in 2 cases 4 samples; in 7 cases 3 samples).

Twenty-one patients with PJI had positive cultures in the sonicate fluid and in 16 of them the same microorganisms were isolated in the periimplant tissue. Table 2 shows the microorganisms isolated in 21 patients with PJI. The sensitivities of sonicate fluid (≥20 CFU) and peri-prosthetic tissue culture for the detection of PJI were 87.5 % (21/24), (95 % CI, 67.4−97.3 %) and 66.7 % (16/24) (95 % CI, 44.7−84.4 %), *p* = 0.09, respectively. Specificities were 100 % for both techniques (95 % CI, 97.9−100 %). In the PJI group, *Staphylococcus* spp. was the most frequent microorganism isolated in both sonicate fluid and peri-prosthetic tissue cultures, 77 % (17 out 22) and 88.2 % (15 out 17), respectively. We did not detect different morphological features, including SCV, and all isolates from synovial fluid, periprosthetic tissues and sonication fluid showed a normal phenotype.

**Table 2:** Microbiology results in 21 patients with definitive PJI and positive cultures

Case	Peri-implant tissue	Tissue culture-number positive/submitted	Sonicate fluid (>20 CFU)	Colony forming units	Histopathology
1	*S. lugdunensis*	1/4	*S. lugdunensis*	5	+
2	*S. lugdunensis*	3/3	*S. lugdunensis*	>100	+
3	*S. epidermidis*	2/3	*S. epidermidis*	>100	+
4	*S. epidermidis*	3/4	*S. epidermidis*	>100	_
5	*S. epidermidis*	2/3	*S. epidermidis*	>100	_
6	*S. epidermidis*	2/4	*S. epidermidis*	>100	+
7	*S. epidermidis*	2/3	*S. epidermidis*	>100	_
8	*S. epidermidis*	3/3	*S. epidermidis*	>100	_
9	*S. epidermidis*	3/3	*S. epidermidis*	>100	_
10	*S. epidermidis,*	3/3	*S. epidermidis,*	>100	_
	*S. haemolyticus*		*S. haemolyticus*	>100	
11	*S. schleiferi*	2/3	*S. schleiferi*	>100	+
12	*S. capitis*	3/3	*S. capitis*	60	_
13	*S. capitis*	2/3	*S. capitis*	>100	_
14	*E. cloacae*	2/2	*E. cloacae*	>100	+
15	*S. hominis*	4/4	*S. hominis*	>100	+
16	*Brucella abortus*	3/3	*Brucella abortus*	>100	+
17	--	0/3	*S. epidermidis*	>100	+
18	--	1/3	*S. epidermidis*	>100	+
19	--	0/3	*E. faecalis*	>100	+
20	--	0/3	*Micrococcus* ssp	55	+
21	--	0/4	*Brevibacterium* ssp.	>100	+

*PJI* Prosthetic joint infection, *CFU* Colony-forming unit

### Outcome

The median follow-up of the 198 patients was 36 months (IQR: 21 to 46 months). During follow-up 9 (37.5 %) patients out of 24 in the PJI group had implant failure whereas in the 174 within the AL group, only 1 patient (1.1 %) underwent repeat revision arthroplasty due to a related-implant infection (*p* < 0.0001).

Five (55.5 %) patients with PJI and implant failure had more revision arthroplasties during the first year after the last implant placement than those patients with PJI without implant failure (1 patient; 6.7 %) (RR 3.8; 95 % CI 1.4-10.1; *p* = 0.015).

Six (25 %) patients finally diagnosed of PJI were initially diagnosed of AL in the first year after primary arthroplasty, whereas it was only 16 (9.2 %) patients in the group of true AL (RR 2.7; 95 % CI 1.2-6.1; *p* = 0.03).

Patients with PJI and implant failure received more antibiotic treatment than those with PJI without implant failure (p < 0.001). None of the 174 patients with diagnosis of AL received antibiotics beyond standard peri-operative prophylaxis while 11 of the 24 (45.8 %) patients with diagnosis of PJI received antibiotic therapy post revision arthroplasty *p* < 0.0001. Six of these 11 patients (54.6 %) who received antibiotics post revision had implant failure whereas only 3 of 13 patients (23,1 %) with PJI who did not receive antibiotics had implant failure (RR = 2,4; 95 % IC 0.8-7.3; *p* = 0.11). Additionally, 8 of 9 patients with PJI and implant failure required of a new surgery: two-stage exchange revision was the most frequent type of surgery in 5 cases (55.5 %) followed by surgical debridement in 2 cases (22.2 %) and Girdlestone procedure in 1 case (11.1%). The other patient with implant failure was under chronic suppressive therapy (Fig. 1). The highest functional status was achieved in PJI patients with implant failure who underwent two-stage revision 5/5 (100 %) whereas the other 4 patients with PJI and implant failure who underwent other procedures (debridement in 2 cases, suppressive therapy in 1 case and Girdlestone in 1 case) only 1 case (suppressive therapy) achieved good functional status *p* = 0.02.

Only 1 of the 4 excluded patients (the patient with intraoperative pus in the revision arthroplasty) died, but the death was unrelated to the surgery.

## Discussion

In the present study we analysed the incidence of PJI among patients with pre-and intra operative diagnosis of AL and if the diagnosis of PJI, according to microbiological and histopathological findings, was predictive of implant failure, including reinfection.

Almost one decade ago, the National Institutes of Health Consensus Development Conference on Total Knee Replacement suggested that it was critical to identify the cause of the original prosthesis failure [12]. However, in the decade that has followed, the diagnosis of orthopedic implant infection remains challenging, and is probably one of the reasons that the pathogenesis of AL is poorly understood. The possibility that bacteria can live on/and around foreign bodies leading to implant failure with no signs or symptoms of infection could be rationalized in some cases of AL [13]. Several investigators have used different sonication regimens as a biofilm-sampling strategy from orthopedic implants removed for presumed AL. Dobbins et al. [14] showed positive sonicate cultures in 77 % of removed orthopedic fixation devices and Moussa et al. [15] in 52.3 % of patients with removal of fracture-fixation hardware. Our results are in close agreement with those of the most recent studies [5, 6, 16] we found positive sonicate cultures in 10.6 % (21 of 198) of patients. In this respect, a new published study recommends implant sonication in patients with delayed orthopedic implant failure with no clear of signs of infection [17].

We found a discrepancy between pre-and intra operative suspicion of AL and the definitive diagnosis. Intraoperative cultures results (sonication and/or peri-implat tissue) with or without histopathology findings determined that a total of 12.1 % of patients had a diagnosis of PJI, 37.5 % of whom had implant failure. When the diagnosis of PJI was made by combining sonication, peri-implant tissue culture and histology, it was possible the diagnosis of 8 (33.3 %) PJI cases, 6 (75 %) of which developed implant failure. This is an important finding, because the conjunction of histology and positive cultures (periimplant tissue and sonicate fluid) was mainly associated with failure of the prosthesis. Our sonication technique was based on previous studies performed on hip, knee, shoulder and spine implants with similar biofilm-sampling protocol that involved a concentration step [5, 10, 11]. We showed that the sensitivity of sonicate fluid culture (CFU ≥20), which requires a single specimen, was more sensitive than peri-prosthetic tissue culture in detecting PJI. Some have questioned the real cutoff value for sonicate fluis cultures [18]. In the present study, all positive sonicate fluid cases had ≥55 CFU and 84.6 % had ≥100CFU. It suggests that these positive sonication results represent true-positive results. However, in 2 of our PJI cases, sonication cultures identified unusual organisms, *Micrococcus* spp. with 55CFU and *Brevibacterium* spp. with >100 CFU, respectively. They did not receive specific antimicrobial therapy aside from routine prophylaxis suggesting that the managing physicians considered a non-pathogenic role and both had favorable outcomes. Thus, some of these cases, spite of the presence of positive histology, could well have been false-positive intra-operative cultures.

Antibiotic therapy post-revision surgery in patients with PJI was no related to a better final outcome. There were no statistically significant difference in functional status between PJI patients with antibiotic post-revision and non-treated patients *p* = 0.61. In fact, patients who developed implant failure had been previously treated with antibiotics more frequent than patients without implant failure. PJI patients with implant failure and two-stage revision had the better outcome. This type of surgery is the gold standard for chronic PJI and offers more than a 90 % chance of eradication of the infection [19–21]. Most of PJI patients treated or not with antibiotic therapy, showed favorable outcomes to the prosthesis-related issues. However, longer follow up may be needed to detect recurrences. Finally, we observed in agreement with Ribera et al. [22] a correlation between prosthesis-age and risk for implant failure, which supports that early loosening is more often caused by hidden PJI than late loosening.

This study has several limitations. Firstly, a gold standard is lacking for diagnosis of implant-associated infection in cases of revision arthroplasty due to AL. Secondly, the pre or peri-operative synovial fluid leukocyte count was not performing. Thirdly, due to the small sample size of PJI infections, the study, it may lack the statistical power to detect some associations. Despite the limitations of the study, our patients were classified according to strict criteria (correlation of clinical, histopathological, and microbiological findings) and according to the low failure rate after primary total hip or knee arthroplasties (1−5 %).

## Conclusions

In summary, we found discrepancy between pre-and intra operative suspicion of AL and postoperative diagnosis. A positive histology and peri-implant tissue and sonicate fluid cultures could predict patients with PJI and implant failure. Patients with partial hip revisions for a presumed AL had more risk of PJI. There was a correlation between prosthesis-age and risk of implant failure, which supports that early loosening, is more often caused by hidden PJI than late loosening.

## Abbreviations

PJI: Prosthetic Joint Infection
AL: Aseptic Loosening
CFU: Colony Forming Units
